# Feasibility and acceptability of brief individual interpersonal psychotherapy among university students with mental distress in Ethiopia

**DOI:** 10.1186/s40359-021-00570-1

**Published:** 2021-04-27

**Authors:** Assegid Negash, Matloob Ahmed Khan, Girmay Medhin, Dawit Wondimagegn, Clare Pain, Mesfin Araya

**Affiliations:** 1 Department of Psychiatry, College of Health Sciences, School of Medicine, Addis Ababa University, Addis Ababa, Ethiopia; 2 Department of Psychology, College of Education and Behavioral Sciences, Wolaita Sodo University, Wolaita Sodo, Ethiopia; 3 Aklilu Lemma Institute of Pathobiology, Addis Ababa University, Addis Ababa, Ethiopia; 4 Department of Psychiatry, University of Toronto, Toronto, Canada

**Keywords:** Mental distress, Feasibility, Acceptability, Interpersonal psychotherapy, University students, Ethiopia

## Abstract

**Background:**

The prevalence of mental distress among university students in low- and middle-income countries (LMICs) is increasing; however, the majority do not receive evidence-based psychological intervention. This calls for the provision of culturally adapted psychological therapy in higher education institutions in LMICs. The aim of this pilot study is to evaluate the feasibility and acceptability of Interpersonal Psychotherapy adapted for Ethiopia (IPT-E) among Wolaita Sodo University students and to assess the preliminary outcomes of IPT-E in reducing symptoms of mental distress and in improving functioning.

**Methods:**

We used a quasi-experimental single-group pre-post-test study design. As indicators of feasibility of IPT-E, we used consent, treatment completion and attrition. We used Client Satisfaction Questionnaire and semi-structured interview to measure the acceptability of the intervention, self-reporting IPT-E checklist to assess treatment adherence and World Health Organization Disability Assessment and Self-Reporting Questionnaire-20 tools to assess functional impairment and mental distress, respectively. We used percentage, frequency, mean and standard deviation to summarize the demographic variables, feasibility and acceptability of IPT-E. We analyzed changes from pre- to post-tests of mental distress and functioning results using paired t-test and Wilcoxon signed-rank tests. Independent sample t-test and one way-ANOVA used to assess the difference in mean score of in demographic variables at baseline and eight weeks. The qualitative data was analyzed with the support of open code 4.02.

**Results:**

IPT-E was feasible (consent rate = 100%; completion rate = 92.31%; attrition rate = 7.69%; mean score of the sessions = 8 and mode of the session = 8). The total mean score of treatment satisfaction was 27.83 (SD = 4.47). After the delivery of IPT-E, symptoms of mental distress were decreased, functioning was improved and therapist adherence to the treatment model was 100% (i.e. treatment delivered according to the IPT-E guideline).

**Conclusion:**

IPT-E was feasible and acceptable to treat university students with mental distress in low-income country setting. The preliminary results also suggest promising viability of IPT-E in higher education institutions of low-income country setting for students with symptoms of anxiety and depression.

**Supplementary Information:**

The online version contains supplementary material available at 10.1186/s40359-021-00570-1.

## Background

The prevalence of mental distress understood as the symptoms of anxiety and depression is higher among university students as compared with the general population [1] has increased over the past few years [2]. For instance, the prevalence of depression symptoms among university students is 30.6% worldwide [3], 24.4% in low- and middle-income countries (LMICs) [4] and the pooled prevalence of anxiety and depression is 37.73% among Ethiopian university students [5]. Globally reported risk factors of experiencing mental distress among students are: (i) academic pressure and financial constraints, [1, 6]; being away from home for the first time and starting new peer relations [7]; inadequate social support [8, 9]; (iv) loneliness [10]; (v) substance use [11]; (vi) earlier age of onset of symptoms [12], where most of university students’ age range from 17 to 25 years [13]; and (vii) interpersonal conflicts [14].

Increased severity of mental distress along with the lack of access to professional mental healthcare are associated with an adverse effect on students’ academic achievement, physical health, emotion, self-esteem, social relationships, cognitive development, and their overall quality of life. Evidence showed that students with mental distress and who did not receive mental healthcare timely experienced low academic performance [15] and suicidal thoughts and attempts [16]. A Previous study reported that college students who experienced depression and sleep disturbance in America had a high burden of comorbid anxiety and poor mental and physical functioning [17]. The burden and functional disabilities of mental distress are more severe in LMICs university students [7], where the accessibility of psychological interventions is limited.

Despite the increasing number of universities in LMICs and a growth in the proportion of students enrolled in these universities, the provision of professional mental health services for students remains underdeveloped, largely because there are insufficiently trained therapists/counselors [18]. As a result, the majority of the students in LMICs do not receive professional mental health care, even if the students request or require it [19]. Most students in need receive support from informal sources such as friends, family, traditional healers, and religious leaders [20]. In some settings in LMICs the inability of students to receive formal mental health services may be associated with an increase in the severity of mental distress and the development of Common Mental Disorders (CMDs), withdrawal from the university, substance use, self-harm and suicide, low self-esteem, isolation, and poor academic performance [21–24]. In so far as the prevalence and burden of anxiety and depression are rapidly increasing among students, universities are advised to ensure adequate student counseling services to prevent and treat student for distress and mental health symptoms [25]. Early detection of mental distress and feasible psychological interventions are needed in LMICs universities, to combat the negative impacts of mental distress on education performance, social interactions, health, and functional impairments of students [10, 26].

Psychological interventions are effective in treating CMDs (anxiety and depression) in LMICs [27] and are recommended by the World Health Organization intervention guide [28]. When employed they result in fewer relapses and premature treatment termination as compared to pharmacotherapy for students with depression [29]. Of the potential psychological interventions, Interpersonal Psychotherapy (IPT) is effective in resolving symptoms of depression and anxiety and improving interpersonal relationships and has been used in primary health centers in LMICs, including Kenya [30], South Africa [31]**,** Egypt [32], and Ethiopia [33]. IPT is an evidence based brief time-limited manualized therapy, which is used to treat clients who struggle with depression associated with current interpersonal dispute/conflict, role transitions/life changes, grief/loss and social isolation/loneliness [34].

Interpersonal conflict is defined as “a situation in which the patient and at least one significant other person have non-reciprocal expectations about their relationship” [34] informally understood as disagreements, arguments and disputes. During the university stay, students who experience conflict with their friends and family increases their likelihood of developing mental distress [14, 35]. Interpersonal conflict is chosen as an IPT focus area when worsening symptoms are connected to disagreements and arguments. Role transition is focal area of IPT that is defined as an individual who is unable to adapt to new life changes (both positive and negative) that include moving away from the family, poverty, separation or rejection by a lover, caring for someone who is dying, serious illness and getting marriage [34]. All university students face role transitions and it is chosen as an IPT target when worsening symptoms are linked to significant life changes with challenges to adapt to new circumstances.

Grief is another IPT focal area that occurs when a student loses a significant person by death [36]. It is common to experience grief reactions such as sadness, feelings of discomfort, guilt and anger for most people across the world, but if it fails to resolve within a reasonable time dictated by local cultural expectations, it increases the risk for CMDs and it negatively affects students’ academic performance and quality of life [36–38]. Besides, prolonged grief impairs participation in social or enjoyable activities, deprives mood and deny the death of the closed person [39]. The last IPT focus area is social isolation, which is associated with the person talks about feeling lonely and separate from others that are caused by problem of maintaining relationships with friends, family, relatives or others [34].

However, the feasibility and acceptability of IPT has not been well studied in LMIC university setting, where most students’ mental distress is mainly caused by role transitions, interpersonal conflicts and grief. At the global level, some studies showed the feasibility and acceptability of IPT among adolescents. For example, a quasi-experimental study conducted in Columbia reported that brief IPT was feasible and acceptable in reducing mild to moderate symptoms of depression and improving social functioning among adolescent students [40]. A systematic review and meta-analysis also reported that IPT was an effective therapy to treat adolescents with symptoms depression [10, 41]. Furthermore, an experimental study conducted among Iranian university students reported that students who received brief group IPT showed a significant reduction in depression symptoms compared to the control group [42]. However, a study conducted in Australia reported that individual IPT is more effective than group IPT for treating depressed adolescents at school setting [43]. Likewise, another evidence showed that the majority (95%) of the college students prefer individual psychotherapy to group counseling [44].

The high prevalence of mental distress and challenges encountered by university students in LMICs are a call to implement culturally appropriate evidence-based and practical psychological intervention. Although previous studies recommend the need for accessible and acceptable evidence-based mental health interventions for university students with mental distress, to our knowledge, there is no published evidence from studies conducted in Ethiopia on the feasibility and acceptability of IPT for university students with mental distress. The primary aim of this pilot study was to evaluate the feasibility and acceptability of brief individual Interpersonal Psychotherapy adapted for Ethiopia (IPT-E) among Wolaita Sodo University (WSU) students. And the secondary objective was to evaluate outcomes that include the preliminary effectiveness of IPT-E in reducing symptoms of mental distress and improving functioning.

## Methods

### Study area and context

The current study was conducted at WSU, a non-profit public university in Wolaita zone, Wolaita Sodo City which is located 320 km south of Addis Ababa, the capital of Ethiopia. The University was established in 2007 with intake of 801 students in four faculties and sixteen departments. Currently, the University runs undergraduate and graduate programs in regular, weekend and summer courses in six colleges and five schools with a total student population of 30,000. The University has its own health service facilities and it has four full-time counselors with four counseling offices that provide free counseling services for students with mental health and psychosocial problems. WSU has a referral teaching hospital (named as Otona) that provides health care services, including psychiatric care for the surrounding community and students. A dedicated clinic within the university compound provides health care services for students and it has a referral system with the hospital for students with severe physical and mental illnesses. In response to the occurrence of coronavirus (COVID-19) pandemic the University has established a Mental Health and Psychosocial Support center to help students with COVID-19 related issues.

### Study design

We used a quasi-experimental single group pre- and post-test design with repeated measures for secondary outcomes. The data were collected from December 2019 to February 2020.

### Study participants and their recruitment procedures

Our source population is undergraduate students in WSU. We posted flyers in target areas, including: the students’ dormitories and cafeteria, the main gate of the university, the library, and student’s health clinic. In addition, we prepared three banners in collaboration with the WSU student’s Dean and counseling offices. These banners were posted in the same strategic areas as the flyers. The flyers and banners were written in Amharic, the official language of Ethiopia which most students speak. The flyers and banners encouraged those students who experienced symptoms of psychological distress to come to student services for IPT-E. The flyers and banners included the names, addresses and contact data of the counselors as well as their time of availability.

**Fig. 1:**
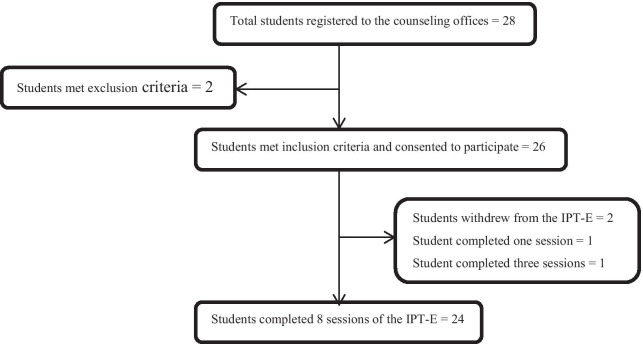
Flow of study participants

The counselors registered students that came to the counseling offices requesting assistance following the distribution of the flyers and erection of the banners, and screened them based on eligible criteria for IPT-E: they were an undergraduate student; scored 8 or more on SRQ-20; they were 18 years or older; willing to attend at least 4 IPT-E sessions and able to speak the Amharic or Wolaitigna or Afan Oromo language. The exclusion criteria were: students with serious physical illnesses; suffering from cognitive impairment; severe mental illness; and already receiving psychiatric medication or psychological treatment or traditional treatment. The counselors asked the students some questions to identify these exclusion criteria. Twenty six eligible students were recruited and participated in the IPT-E sessions. See Fig. 1.

IPT has been adapted to the Ethiopian context in collaboration with Toronto Addis Ababa Psychiatry Project and the Biaber Project and named Interpersonal Psychotherapy adapted for Ethiopia (IPT-E) [33]. This culturally adapted talk therapy is a brief manualized evidence supported intervention used to treat clients with CMDs caused by loss, life transitions/role changes, and interpersonal conflicts [33]. Social isolation was not considered as focal area of IPTE, because in a country where everyone lives in a community, the issues of social isolation and IP deficit are rare foci for symptoms. The IPT-E manual has 8 modules, 4 that contains a basic and interactive orientation to mental health in Ethiopia, including mental health service delivery, mental health risks and resilience, the therapy relationship, safety issues, mental health screening, and 4 modules on the IPT-E: beginning phase, middle phase, termination phase, using relevant example cases and includes an evaluation of IPT-E training outcomes. In addition, the manual has Amharic language case-based training videos.

The IPT-E beginning phase includes screening a client for mental distress; how to select cases appropriate for IPT-E; ensuring consent to participate in the treatment; and screening for client safety such as suicide, domestic violence, harming others and substance use. The counselors understand clients’ problems, symptoms, functioning, and explanatory model using a Treatment Tracking Form (TTF). They also conduct an interpersonal inventory, and provide tailored psycho-education for the client. Finally, the counselors formulate one or maximum two IPT-E focus area/s (grief, life change or disagreements) for the next middle phase of the intervention.

The middle phase of IPT-E is the heart of the intervention, where general techniques such as role play, psychosocial supports, brainstorming, and communication analysis were practically implemented on the selected IPT-E focus area/s. This occurred in combination with several techniques to address the focus area specifically. In this phase, symptoms and functioning are tracked at each session using the TTF. In the termination phase, the counselor reviews the client’s efforts, progress, therapeutic achievements and changes made in the beginning and middle phases. Throughout treatment the counselor has an IPT-E treatment checklist to note the various IPT-E activities used in the counseling process.

The IPT-E was conducted on a weekly basis and lasted for 40–60 min per session. The number of sessions attended in the IPT-E ranged from 4 to 8. IPT-E has a screening tool which consists of 6-items used to identify participants with mental distress. Both the screening and treatment were given by four counselors under the close supervision of the principal investigator in their own counseling offices, which were well furnished, ventilated, and attractive. All the counseling offices are situated in the same building as the student’s clinic, which promoted a robust referral system and avoided stigma of student clients having separate mental health services. The sessions were not audio-recorded because clients were not comfortable with this. However, the delivery of IPT-E in every session was documented by the counselors using the IPT-E treatment checklist. All counselors had their clients’ cell phone numbers to remind them of the time of their appointment. Clients who had to miss a session due to unexpected events agreed in advance to communicate his/her counselor via cell phone and then rebook for the next appropriate day. The first author of the article supervised the counselors’ work every week.

### IPT-E training for counselors

The intensive IPT-E training was given for four consecutive days and included practical sessions on the clinical skills required. Initially, five counselors participated in the training, but four completed the training. The two women counselors had a Bachelor degree in Public Health, one male counselor had a Master’s degree in Public Health and the other had a Master’s degree in Counseling Psychology. Three of them could speak Amharic and Wolaitigna languages fluently and one counselor could speak Afan Oromo and Amharic languages fluently. The training was given by the principal investigator who has a Bachelor’s degree in Psychology, a Master’s degree in Counseling psychology, had 2 years counseling experience in WSU counseling office and has attended the theoretical and practical training of IPT-E given by the manual adaptors.

To evaluate the outcome of the training, the trainer administered pre- and post-tests prepared from the IPT-E manual and a training satisfaction feedback measuring tool. All counselors showed a significant change in the post-test result compared to the pre-test indicating that the counselors received the necessary knowledge to implement IPT-E in the practical session. The practical sessions also helped them to acquire the clinical skills of implementing IPT-E. Besides this, all the counselors rated the training as excellent and reported that the standard quality of the training was high and fulfilled their expectations. They reported that they received practical knowledge that enabled them to treat students with symptoms of anxiety and depression.

### Measures

Five instruments were used to collect the demographic and outcome data. IPT-E screening tool and TTF were used together to collect data including participants’ age, sex, marital status, perceived cause of the problem, treatment received, onset of the problem, concurrent problems, medication, substance use, experience of gender based violence, suicide ideas, plan and past attempts, and thoughts and past attempts of harming others. The second instrument was IPT-E feasibility measuring tool, which was used to evaluate the number of students who attended the intervention session, consent rate, completion rate, attrition rate, the duration of the intervention session attended, and the mean and modal number of sessions completed over 8-weeks.

The third instrument was Client Satisfaction Questionnaire-8 items (CSQ), which was used to measure acceptability of IPT-E. The 8-items were rated on a Likert scale ranging from 1 to 4 that yield a minimum total score of 8 and a maximum total score of 32 [45]. In addition to this, a semi-structured interview was conducted with 10 clients to explore further satisfaction received from the intervention. Redundancy of the clients’ response to the open ended questions framed from CSQ indicated the saturation of the data. The qualitative data were audiotaped. The fourth was fidelity to the IPT-E treatment that included intervention adherence and the number of session attended (dose) [46]. This treatment adherence was assessed by the IPT-E treatment self-report checklist, which was coded as “yes” or “no” (“yes” indicates the accomplishment of the expected key tasks within each session and “no” represents a failure to accomplish the expected key activities in each counseling session). The counselors completed the checklist for every session of the IPT-E.

The fifth tool was World Health Organization Disability Assessment (WHODAS-2.0) [47]. This instrument is a self-administered 12-item scale designed to measure functional difficulties caused by mental distress in the past 30 days. It has six functional domains which are: understanding and communicating, getting around, self-care, getting along with people, life activities, and participation in society with a Likert scale ranging from 1 (none) to 5 (extreme/cannot do). Scores are computed either by adding the response of items in each domain separately or by adding all the responses together to get a global score. A higher score indicates greater functional impairment. WHODAS-2.0 has been adapted and validated in Ethiopia for people with severe mental illness [48].

The six instrument was Self-Reporting Questionnaire-20 items (SRQ-20) used to measure the extent of mental distress in the last 30 days. This tool is primarily developed by WHO and recommended to LMICs to screen positive cases of mental distress [49]. It has binary options (yes = 1 indicating the presence of the symptom, no = 0 indicating the absence of the symptom). Adding each item gives a maximum total score of 20″ [50]. The SRQ-20 has been validated in Ethiopia with different cut-off points [51, 52]. For the present study, we used a cut-off point 8 or above on SRQ-20 to identify positive cases to mental distress based on a previous validation study [53].

See Additional file 1.

### Data analyses

In the present study, data were collected at baseline and at eight-weeks. The data collected using SRQ-20 and WHODAS-2.0 were measured twice, whereas the data measured by CSQ, IPT-E feasibility measuring tool, and interviews of the clients were collected at eight weeks. The quantitative data were analyzed using Statistical Packages for the Social Sciences version 20 after cleaning, checking the missing values, outliers, and after checking normality assumption of the distribution of continuous variables. The demographic variables, feasibility and acceptability of IPT-E and treatment satisfaction were summarized using percentage, frequency, mean, and standard deviation.

The preliminary effectiveness of IPT-E measured by SRQ-20 at pre- and eight weeks were analyzed using paired t-test and the functioning data measured by WHODAS-2.0 at baseline and post-assessment were analyzed by Wilcoxon signed-rank test. Independent samples t-test and one-way ANOVA were used to compare the mean score difference of two and more categorical variables with normally distributed continuous outcomes, respectively. The effect size was interpreted based on 0.01 = small effect; 0.06 = moderate effect; and 0.14 = large effect [54]. Statistical significance was reported whenever *p*-value was less than 0.05. The audiotaped qualitative data of the acceptability of IPT-E were transcribed verbatim and translated and then analyzed using thematic analysis approach supported with a qualitative software, open code 4.02 [55].

### Ethical issues

All the ethical considerations and the methodological plausibility of the current study were done according to Addis Ababa University College of Health Sciences (AAU-CHS) Institutional Review Board [28] guideline. Ethical clearance was obtained from the IRB of AAU-CHS with protocol number of 045/17/Psych. The intervention was initiated after receiving written informed consent from all study participants. The participants were informed that they could withdraw from the study at any time if they were not comfortable participating in the study, without prejudice. There was a referral system through the students’ clinic to the Otona hospital for those students who met the exclusion criteria of the present study. As a result, two students who were previously diagnosed with severe mental illness and taking psychotropic medications were referred to the Otona hospital for further psychiatric care. Finally, the collected data were kept anonymous and confidential throughout the whole study process by locking the computer using a password known only by the principal investigator. The data documented on the hard copies were kept in locked box of the principal author’s house.

## Result

### Demographic characteristics of the study participants

The proportion of male–female participants was equivalent (50%) and 79.2% were single. Their age ranged from 18 to 23 years with a mean age of 21 years (SD = 1.49). The majority (75%) perceived that the causes for their mental distress were disagreement (conflicts with family, friends and dorm mates and life changes (being new to university life, separation or rejection by a lover and loss of support/economic hardship). All participants were not receiving treatment from traditional healers or from other sources throughout the study period and 50% reported that their distress started before two years prior to this study. All participants reported that they did not have concurrent medical conditions such as HIV, TB, malaria, diabetes and heart disease and substance use. They were not taking any medication during the intervention period; they reported no previous experience of gender-based violence and no past attempts of suicide and harming others. The majority (62.5%) had no thoughts of suicide and 95.8% had no plan to commit suicide. In the past month the majority (75.0%) had no thoughts of harming others (Table 1).

**Table 1:** Demographic characteristics of the participants (n = 24)

Variable	Number	Percent
Marital status		
Single	19	79.2
Married	2	8.3
In relationship	3	12.5
Age		
Mean (Standard deviation)	20.67 (1.49)	
Perceived cause of mental distress		
Grief	3	12.5
Conflict	9	37.5
Life change/role transition	9	37.5
Conflict and life change	2	8.3
Loss and life change	1	4.2
Did not received treatment from traditional healer during the study period	100	100
Problem has been present continuously for 2 or more years		
Yes	12	50
Had ideas of suicide in the past month		
Yes	9	37.5
Had plan of suicide in the past month		
Yes	1	4.2
Had thoughts of harming others in the past month		
Yes	6	25.0
Is the client fit for IPT-E?		
Yes	24	100

### Feasibility of IPT-E

All participants provided written consent to participate in the IPT-E. Of the 26 eligible participants who initially participated in the intervention, 24 completed the full eight-session treatment resulting a completion rate of 92.31%. The remaining two withdrew from the intervention (one participant attended session one and the other attended three-sessions), which yielded in the attrition rate of 7.69%. These two participants were excluded from the data analysis. The mean, median and mode number of the intervention sessions attended were 8.

### Acceptability of IPT-E

The total mean score of client satisfaction received for IPT-E was 27.83 (SD = 4.47; range = 12–32). The majority (66.7%) rated the quality of the intervention they received being excellent; they qualitatively expressed their satisfaction as follows:

A 21-year-old female student said:

“Ok, thank you, eh…the care that I received is like  translated into English as changing a dried tree to a tree with wet leaf. I received my previous identity; I am confident, strong, have a good communication with my friends (my friends are surprised by my improvement) and I am also happy.” (Interviewee #07).

Another 20-year-old male student explained that:

“If I am not able to get her (the counselor) care, I would be a daily laborer and below my friends, because my family cannot afford private college fees to teach me. God showed me the counselor because she saved me the whole of my life. I found it very helpful.” (Interviewee #05).

Forty-six percent of the study participants reported that they definitely received the kind of the services they expected and almost all of their needs were met. Despite this, most students qualitatively expressed the treatment they received as it was beyond their expectations that fully addressed their mental health care needs.

A 21-year-old female student said:

“Normally, when I came to this office, it was because of my friend’s advice. I was not expecting to get such kind of service; I just came to tell my  (translated as internal turmoil) if somebody is ready to listen to me; however, what I was thinking was completely different from what I received. It is beyond my expectation.” (Interviewee #07).

The majority (75.0%) of the participants reported that they would definitely recommend the intervention received to their friends in need of similar care. Over half (54.20%) reported that they were very satisfied with the intervention they received. They qualitatively expressed that all of them were satisfied by the treatment given to them and they would recommend for their friends with mental distress in need of similar mental health care.

A 23-year-old male student said:

“If I love him (friend), why not bring him here? Because I’m satisfied with the service I received… (laugh). Our prophet said that one person is said to be not believed in Islam until he loves what he loves for his brother. Because I am satisfied with the therapy; I am happy to bring my friends who have a problem, even anyone on the street suffering from such a problem if it is not adding a burden on the counselor. Normally, I was laughing a false laugh before therapy, however, now I am normally happy even when I did not score a good exam result.” (Interviewee #01).

Another participant stated:

“Yes, I received important advice that not only helps me but also enables me to even advise someone else; now I can advise another person who has similar feelings of distress. The interviewer asked, you mean you can replace the counselor? Yes, laugh…laugh… I can send other people with a problem by informing them there is a counselor who provides counseling services and I can bring them here.” A 20-year-old male interviewee #04.

Most (70.80%) participants reported that the mental health service they received helped them effectively deal with their problem and half (50%) of them rated their overall treatment satisfaction as mostly satisfied or very satisfied.

A 23-year-old male student said:

“Extremely, my face speaks; I am very happy today; my previous happiness has returned again; I started to communicate with many people. Previously even I do not know the name of the students in our class; I asked my friend who was this student in our class? You know I entered to the class before anyone and sat at the back corner and I left the class at the end when all students went out. But now, I play and communicate with the students; we go together up-to the dormitory. I have a good relationship with my dorm mates; there is a student who disturbs me in the dormitory; I tolerate him very well; if I were in my previous mental state, I would have conflict with him.” (Interviewee #01).

Most (62.5%) participants reported that they would definitely return to the counseling office if they need care again.

22-year-old male student explained:

“I hope I would not have to face the problem, but if it occurs again, I will come back.” (Interviewee #08)

There was no statistically significant difference in any of the demographic variables in the treatment satisfaction.

### Fidelity

The majority of the participants attended 8 IPT-E sessions. Each session lasted 40 to 60 min as is recommended by the IPT-E guidelines. In addition to the provision of training for the counselors and regular supervision of the delivery of IPT-E counseling service, the accomplishment of the key activities to be done in each session were immediately evaluated. All counselors ticked “yes” for all activities, indicating that the necessary tasks were done in each of the IPT-E sessions.

See Additional file 2.

### Outcome evaluation

The IPT-E showed preliminary effectiveness in decreasing symptoms of mental distress and improving functioning among students. There was a statistically significant decrease (*p*-value = 0.001) in mean score symptoms of mental distress from baseline (M = 14.13, SD = 3.70) to eight weeks post assessment (M = 3.21, SD = 3.12) with effect size of 0.89 (large effect). At the post-assessment, four participants (16.6%) scored above the cut-off 8 on SRQ-20 (2 participants scored 10 points and another 2 participants scored 14), even if they showed symptoms reduction in mental distress from the baseline.

The data collected through Treatment Tracking Form (TTF) showed an improvement of mental distress in each session. Likewise, there was reduction in the frequency of mental distress symptoms from pre-to-post assessment in the past two months. During the pre-test a large proportion of the participants reported that they experienced symptoms such as being sad, miserable or hopeless (46%); had little pleasure in doing things (54%); felt anxious (67%); and experienced a lack of energy on several days in the past one month (50%). Of all participants, 29% felt restlessness and 38% had trouble concentrating on conversations or reading nearly every-day in the past month. Of the total participants, 33% felt bad about themselves and unable to control worrying several days or nearly every-day in the past one month and 42% had trouble sleeping nearly every-day in the last month. Furthermore, 46% of the participants felt afraid that something awful might happen without a reason and 50% experienced more than 5 physical symptoms several days in the past month.

After the two months IPT-E intervention, the majority (75%) of the participants had not felt symptoms such as being sad, miserable or hopeless and 71% had pleasure in doing things. The majority (75%) reported that they had no symptoms of anxiety and 79% recovered their energy after the intervention they received. Almost all (91.70%) of the participants felt symptoms such as restlessness and feeling bad about themselves had remitted. They no longer had difficulty concentrating on conversations or reading after the intervention they received. Most (79%) participants no longer experienced problems controlling they worrying and 88% were sleeping well. Finally, the majority (75%) lost their dread of something awful happening without reason and 83% no longer had 5 or more physical symptoms. For further information see Table 2.

**Table 2:** Pre and post-tests percentage of metal distress symptoms

Questions	Pre-test					Post-test			
	Not sure (%)	Several days (%)	Over half the days (%)	Nearly every day (%)	Never (%)	Several days (%)	Over half the days (%)	Nearly every day (%)	Never (%)
Felt sad, miserable, down or hopeless?		45.8	12.5	37.5	4.2	16.7	4.2	4.2	75
Felt little pleasure or interest in doing things?	4.2	54.2	8.3	25	8.3	16.7	4.2	8.3	70.8
Felt nervous/anxious?		66.7	25		8.3	12.5	8.3	4.2	75
Not enough energy, that everything is an effort?	4.2	50	20.8		4.2	8.3	8.3	4.2	79.2
Felt so restless it is hard to sit still?	8.3	25	25	29.2	12.5	4.2	4.2		91.7
Had trouble to concentrating on conversations/reading?	4.2	20.8	20.8	37.5	16.7	8.3	4.2		87.5
Felt really bad about yourself, that you are a failure or that you have let your family down?	12.5	33.3	12.5	20.8	20.8	4.2	4.2		91.7
Felt you could not stop or control worrying?	8.3	33.3		33.3	25	16.7	4.2		79.2
Had trouble sleeping?	8.3	12.5	20.8	41.7	16.7	4.2	4.2		87.5
Felt afraid as if something awful might happen without a reason?	16.7	45.8		20.8	16.7	20.8	4.2		75
How many days have you had more than 5 physical symptoms (e.g. aches and pains, palpitations, burning/numbness/crawling sensations)?	4.2	50	4.2	25	16.7	12.5	4.2		83.3

The overall mean score of WHODAS-2 was significantly improved (*p*-value = 0.001) from baseline (M = 34.33, SD = 10.70) to post assessment (M = 22.71, SD = 8.34) with effect size of 0.43, (large effect). The mean scores of WHODAS sub-scales were significantly improved from baseline to eight weeks assessment. For detail information, see Table 3.

**Table 3:** Functioning at baseline and post-test

Measure	Baseline (n = 24)		8-weeks (n = 24)		Effect size	Z	*P*
	M	SD	M	SD			
*WHODAS sub-scales*							
Understanding communicating	5.83	2.04	4.00	2.17	0.32	− 3.28	0.001
Getting around	5.63	2.14	3.88	1.94	0.33	− 3.34	0.001
Self-care	3.92	2.12	2.83	1.24	0.20	− 2.37	0.02
Getting along with people	5.67	2.71	3.96	2.03	0.21	− 2.45	0.01
Life activities	6.42	2.24	3.38	1.35	0.41	− 3.96	0.001
Participation in society	6.88	1.75	4.67	1.93	0.39	− 3.80	0.001
Total WHODAS-2 score	34.33	10.70	22.71	8.34	0.43	− 4.18	0.001

M = Mean; SD = Standard deviation; Z = Z-value; *P* = *P*-value

A total number of days suffering from mental distress among the participants were reduced following the intervention.

Change in mental distress following the intervention stratified by selected variables.

Baseline mean score of SRQ-20 was significantly influenced by gender, duration of depressive symptoms and reporting thoughts of harming others in the past month. However, at the post-test, mean score of SRQ-20 was not significantly affected by any of the pre-specified characteristics of the study participants. There was no statistically significant difference in the mean score of SRQ-20 by marital status and perceived causes of mental distress both at baseline and post-assessment (Table 4).

**Table 4:** Mean score and standard deviation of selected variables at baseline and post-test

Variable	SRQ-20 score at baseline				SRQ-20 score at 8-weeks			
	M	SD	T/F	*P*	M	SD	T/F	*P*
*Sex*							2.02	0.06
Male	12.58	3.29	2.21	0.04	2.00	3.19		
Female	15.67	3.55			4.42	2.64		
*Has the problem been present continuously for 2 or more years?*							0.58	0.57
No	12.25	3.70	− 2.84	0.01	3.58	3.26		
Yes	16.00	2.70			2.83	3.07		
*Ideas of suicide*							1.07	0.3
No	12.87	3.50	− 2.36	0.03	3.73	3.45		
Yes	16.22	3.15			2.33	2.40		
*Thoughts of harming others in the past month*							− 1.2	0.25
No	13.28	3.51	− 2.08	0.05	2.78	2.96		
Yes	16.67	3.27			4.50	3.51		
*Marital Status*								
Single	14.63	3.85	0.86	0.44	3.26	3.41	0.38	0.69
Married	12.50	0.71			1.50	0.71		
In relationship	12.00	3.46			4.00	1.73		
*Perceived cause*								
Loss	14.33	4.73	0.26	0.90	3.26	5.51	1.57	0.22
Disagreement	14.88	3.89			4.33	2.00		
Life change	13.11	4.08			2.22	3.07		
Disagreement and life change	15.00	0.00			0.00	0.00		
Loss and life change	14.00	0.00			7.00	0.00		

M = Mean; SD = Standard Deviation; T = T-value for t-test; F = F-value for ANOVA

## Discussion

Findings of the current study indicated that IPT-E is feasible and acceptable for university students with mental distress in Ethiopia. All eligible students consented to participate in the intervention and only two of them did not complete the 8-session individual IPT-E intervention. Overall, participants were highly satisfied with the care they received. IPT-E provided a promising preliminary result in Ethiopian University in decreasing symptoms of mental distress and improving functioning of university students.

Most of the study participants completed the 8 weekly sessions, which is comparable with the previous feasibility studies where the majority of depressed adolescents completed brief individual interpersonal counseling [40, 56]. The possible justifications for the feasibility of the IPT-E were: the counselors had received intensive training supported by clinical practice which helped them to engage the students; close supervision by the lead author of this paper; use of a fidelity checklist and the preparedness of the clients to give their counselors advanced notice of their absence. Besides this, our study was conducted in a building where a students’ general medical clinic is located so students are not seen to be attending a mental health clinic. This potentially minimized stigma and increased the likelihood of attending the IPT-E sessions regularly [40]. In addition, the rapport building emphasis of IPT-E also played a role for most participants who attended all 8 sessions [57]. However, two participants withdrew from the IPT-E intervention, because one participant was transferred to a university near to his family and the other was not willing to continue the intervention because he was too busy with school work.

Study participants reported that they were highly satisfied with the intervention they received, indicating that IPT-E was acceptable. A previous study reported that adolescents who received brief IPT were highly satisfied with the care they received [40]. Similarly, in another study adolescents who received 8 sessions of IPT treatment were mostly satisfied/very satisfied with the care they received [58]. As well, the small attrition rate in the present study directly indicates the acceptability of the intervention being provided to the students. The present study has also shown very good treatment adherence what almost all participants completed all 8 treatment session, which is comparable to a prior study in which the treatment adherence of interpersonal counseling by the counselors was reported as good [56]. Along with the educational status of the counselors, the didactic training supported by clinical practice and ongoing clinical supervision enabled the counselors to effectively deliver the IPT-E intervention which is also likely to be linked to the participants’ satisfaction with the rendered counseling service.

The implementation of IPT-E indicated promising results demonstrating a significant decrease of the symptoms of mental distress and by improving functioning of the university students in the study. This finding is consistent with previous studies where most adolescents showed significant improvement in symptoms of depression and social functioning after receiving IPT intervention [40, 43, 56]. Another studies also reported that adolescents who received IPT showed a reduction of depression symptoms as compared to a control group [59, 60]. Similarly, a study in a higher education institution reported that students who received IPT had significantly reduced symptoms of depression compared to a control group [42]. Other reasons associated with the preliminary effectiveness of IPT-E to improve mental distress and functioning are the didactic training and ongoing clinical supervision of the counselors which enabled them to provide IPT-E intervention. As well, ongoing monitoring of the students’ mental distress symptoms and functioning; and the fact that good cognitive capacities [61] and high educational level [62] are associated in the literature with a good response to mental health interventions.

The first onset of most mental health disorders occurs in the age range of 15–25 years [12] and most undergraduate university students are within this age category. As evidence shows, the first-onset of mental health problem can be treated better than the recurrent mental health problem, which might explain why the preliminary effectiveness of IPT-E among the students is high [25]. Other reports note that mild to moderate mental distress among adolescents responds more easily to IPT as compared to severe mental distress [40, 56]. Culturally adapted IPT-E and the high completion of treatment rates of the participants contributed to the improvement of the above mentioned clinical outcomes. This coincide with the results of previous studies where culture tailored mental health interventions enhanced the effectiveness of psychological therapy [40, 56, 60] and the greater number of counseling session attendance predicted the success of mental health intervention [63]. The ideal number of a brief counseling session for anxiety and depression ranges from 6 to 8 sessions [64], which fits with our finding where the mean attendance of IPT-E session was 8. Furthermore, we had taught the counselors during the training and clinical supervision to deliver the counseling service by establishing a good therapeutic alliance and rapport that enhanced the preliminary success of IPT-E in remitting symptoms of mental distress and disability [65].

Limitations: The present study had some limitations: first, our study did not include a comparison group which reduces our confidence in the effectiveness of IPT-E to decrease mental distress and improve functioning. However, the short 8 week time difference between the pre- and post-test in the present study may have addressed the reduction of symptoms by time alone. Second, the small sample size in the present study may limit the generalizability of the findings, so that the interpretation of the clinical outcomes should be undertaken with caution. Finally, in the present study, there were no follow-up sessions. We assessed the counselors’ adherence to the IPT-E manual, but future studies should measure the clinical competency of counselors. A randomized controlled trial of IPT-E and longer follow-up period would secure the evidence base of IPT-Es to further scale-up the service in similar settings.

Despite the drawbacks, the present study has some strengths, including knowledge and skills transferred to the university health workers on how to effectively treat students with mental distress using the IPT-E guidelines. The other strength was that the close clinical supervision of the counselors’ and their adherence to the treatment model increased their knowledge and skills to deliver IPT-E, and extended the capacity of the students’ clinic to address to distress of student. We used locally adapted instruments to screen participants with mental distress and to measure disability. Lastly, we had balanced number of male and female counselors to comfort the need for study participants in a counseling session; we think this paved the way to equalize the sex ratio of participants in the present study.

### Conclusion

The present findings indicate that IPT-E is a feasible and acceptable intervention for the treatment of students with mental distress in low-income country settings. IPT-E also showed promising preliminary effectiveness in reducing symptoms of mental distress and improving functioning of University students. The present study findings provide viable information for mental health service providers in higher education institutions to use and scale-up the manualized IPT-E intervention designed to treat people with CMDs. Therefore, scaling-up of this intervention to the national level and implementing it in higher education institutions in Ethiopia would potentially address the high prevalence and burden of mental distress and reduce the mental health treatment gap among university students [19], although stronger evidence for IPT-E needs to come from randomized controlled trials.

## Supplementary Information

**Additional file 1.** Instruments used for data collection.

**Additional file 2.** Treatment adherence and dose.

## Data Availability

The datasets used and/or analyzed during the present study are available from the corresponding author on reasonable request.

## Abbreviations

LMICs: Low- and Middle- Income Countries
WSU: Wolaita Sodo University
IPT-E: Interpersonal Psychotherapy adapted for Ethiopia
CMDs: Common Mental Disorders
COVID-19: Coronavirus
CSQ: Client Satisfaction Questionnaire
WHODAS-2.0: World Health Organization Disability Assessment
SRQ: Self-Reporting Questionnaire
ANOVA: Analysis of Variance
SD: Standard Deviation
M: Mean
IPT: Interpersonal Psychotherapy
TTF: Treatment Tracking Form
HIV: Human Immunodeficiency Virus
TB: Tuberculosis
AAU-CHS: Addis Ababa University College of Health Sciences
IRB: Institutional Review Board
