# Supplementary material for: Feasibility and acceptability of brief individual interpersonal psychotherapy among university students with mental distress in Ethiopia
Source: BMC Psychol. 2021 Apr 27;9:64. doi: 10.1186/s40359-021-00570-1 (PMC8077191; doi:10.1186/s40359-021-00570-1)
Supplement: Supplementary file 1 — Additional file 1. Instruments used for data collection. [file 40359_2021_570_MOESM1_ESM.docx]

**Title of the manuscript:** Feasibility and Acceptability of Brief Individual Interpersonal Psychotherapy among University Students with Mental Distress in Ethiopia

**Author list:** Assegid Negash^12*^, Matloob Ahmed Khan^1^, Girmay Medhin^3^, Dawit Wondimagegn^1^, Clare Pain^4^ and Mesfin Araya^1^

^1^Department of Psychiatry, College of Health Sciences, School of Medicine, Addis Ababa University, Addis Ababa, Ethiopia

^*^ Correspondence: [assegidn@gmail.com](mailto:assegidn@gmail.com)

^2^Department of Psychology, College of Education and Behavioral Sciences, Wolaita Sodo University, Wolaita Sodo, Ethiopia

^3^Aklilu Lemma Institute of Pathobiology, Addis Ababa University, Addis Ababa, Ethiopia

^4^Department of Psychiatry, University of Toronto, Canada

**Addis Ababa University**

**College of Health Sciences, Department of Psychiatry**

Dear participants of this research, the main objective of the study is to investigate feasibility and acceptability of brief individual interpersonal psychotherapy among university students with mental distress. The study questionnaire has seven parts. Part I focused on assessing some demographic characteristics of the study participants along with screening questions of mental health problems. Part II is Treatment Tracking Form used to measure some mentioned variables. Part III aimed to identify mental distress using an instrument with a cut-off point. Part IV is designed to assess functioning disability using WHODAS-2.0. Part V is aimed to assess treatment satisfaction both quantitatively and qualitatively. Part VI is used to measure feasibility components of the intervention, IPT-E. Part VII is fidelity checklist filled by counselors in each session. Thus, your genuine responses to each items listed in the questionnaires and interview topic guide will help to provide important data for the indented study. Your responses will be kept completely confidential, and will be used for the purpose of this research only. Therefore, be honest to answer the questions and don’t hesitate to ask anything that needs clarification. I would like to thank you for participating in this study.

**Part I. Screening Form for Patients with CMD used in IPT-E**

Reminder: Complete this form and if positive for CMD please continue Treatment Tracking Form

Counseling office name: _____________________________

Counsellor name:_________________________________

Date:___________________________ Chart Number: ______________________

Patient’s cell phone number ____________________________ AGE: ____ Sex: Female Male

Marital status: Married Single Divorced In relationship Other_____________

# of children if: _______# People living in the household: __________Work Type: Student

1. In the last month, how often were you totally unable to carry out your usual activities at home or at work/education, or both, because of any health condition? Record number of days ________
2. In the last month, how often did it feel very difficult to carry out your usual activities at home or at work, or both, because of any health condition? Record number of days ________

| 1. In the last month how often have you: | Not sure | Several days | Over half the days | Nearly every day | Never |
| --- | --- | --- | --- | --- | --- |
| 1. felt sad, miserable, down or hopeless? |  |  |  |  |  |
| 1. felt little pleasure or interest in doing things? |  |  |  |  |  |
| 1. felt nervous/anxious? |  |  |  |  |  |
| 1. not enough energy, that everything is an effort |  |  |  |  |  |
| 1. felt so restless it is hard to sit still ? |  |  |  |  |  |
| 1. had trouble concentrating on conversations/reading/TV etc |  |  |  |  |  |
| 1. felt really bad about yourself, that you are a failure, or that you have let your family down? |  |  |  |  |  |
| 1. felt you could not stop or control worrying? |  |  |  |  |  |
| 1. had trouble sleeping? |  |  |  |  |  |
| 1. felt afraid as if something awful might happen without a reason? |  |  |  |  |  |
| 4.In the last month, how many days have you had more than 5 physical symptoms (e.g. aches and pains, palpitations, burning/numbness/crawling sensations)? |  |  |  |  |  |

5.In the last month, have you had:

1. Ideas or thoughts of suicide? Yes No
2. Plans of suicide? Yes No
3. Past attempts (in your life) Yes No

6. In the last month have you had:

1. ideas or thoughts of harming others Yes No
2. ever hurt anyone physically in the past on purpose? Yes No

(If at risk of harming others contact the supervisor)

Screening indicates treatment is advised? Yes No

PATIENT AGREES to treatment Yes No

If patient declines treatment or is unable to attend PLEASE STATE WHY:

_____________________________________________________________________________________

## Part II. Patient Treatment Tracking Form (TTF)

**REMINDER:** Use this form for patients referred for IPT-E treatment following positive CMD screen. When the patient returns for treatment, retrieve this form and complete it after each session – keep for the mentor/coordinator

**URGENT NOTE/HISTORY**

Session 1 Post screening: Chart number: __________________

Counsellor name:______________________________________ Date:_________________________

1. What does the patient say is wrong with him/her (in their own words)
   _______________________________________________________________________________________________________________________________________________________________________________________________________________________________________________________________
2. What does the patient report as the cause of his/her problem? (in their own words)
   __________________________________________________________________________________________________________________________________________________________________________
3. Does the patient see a traditional healer at the current time? Yes ⬜ No ⬜
4. Recent or Current life stressors that occurred around the time of the onset or worsening of symptoms:
    Loss ⬜ Disagreements ⬜ Life changes / Role transition ⬜
5. Has depression, medically unexplained symptoms and/or anxiety (CMD) been present continuously for 2 or more years? Yes ⬜ No ⬜ Details:___________________________________________________________
6. Other concurrent issues, medical condition or substance abuse (state usage) (please check ALL that are applicable):
   ⬜ Pregnant ⬜ Khat (how much?) ____________________________
   ⬜ HIV/AIDS/TB ⬜ Alcohol overuse (see below)
   ⬜ Cannabis (how much?)________________⬜ Other e.g. malaria, diabetes, heart disease etc Details:_______________________________________________________________________________

_____________________________________________________________________________________

1. Is the patient taking any medications for medical or mental health conditions? Yes ⬜ No ⬜ (if yes please describe)

| Medication name | Dose |
| --- | --- |
|  |  |

8. Has the patient experienced physical or sexual violence

in last year? No ⬜ Yes ⬜ If yes, please write the details ______________________________________________________________________________________

before age of 15? No ⬜ Yes ⬜ If yes, please write the details

______________________________________________________________________________________

ever, in his/her lifetime? No ⬜ Yes ⬜ If yes, please write the details

1. Suicidal NOW?_______________ Previous attempts? _____________Safe for IPT-E? No ⬜ Yes ⬜

Refer acute suicide concern? ______________________________________________________

10. Does the patient over use alcohol? (*more than 10 drinks/week? *and/or has consumed 5 or more standard drinks (the usually accepted size of drink) on any given occasion in the last 12 months? *and/or drinks on average more than 2 drinks per day? *and/or drinks every day of the week?)

No ⬜ Not sure ⬜ Yes if any of the above questions is positive ⬜ (If yes, provide details) ___________________________________________________________________________________

**Part III. Self-Reporting Questionnaire, SRQ-20**

Instruction: The following questions are related to certain pains and problems that may have bothered you in the last one month. If you think the question applies to you and you had the described problem in the last one month, answer Yes. On the other hand, if the question does not apply to you and you did not have the problem in the last one month, answer No. Please, tick your answer on the space provided.

| No | Question | Answer | |
| --- | --- | --- | --- |
|  |  | Yes (1) | No (0) |
| 1 | Do you often have headaches? |  |  |
| 2 | Is your appetite poor? |  |  |
| 3 | Do you sleep badly? |  |  |
| 4 | Are you easily frightened? |  |  |
| 5 | Do your hands shake? |  |  |
| 6 | Do you feel nervous, tense or worried? |  |  |
| 7 | Is your digestion poor? |  |  |
| 8 | Do you have trouble thinking clearly? |  |  |
| 9 | Do you feel unhappy? |  |  |
| 10 | Do you cry more than usual? |  |  |
| 11 | Do you find it difficult to enjoy your daily activities? |  |  |
| 12 | Do you find it difficult to make decisions? |  |  |
| 13 | Is your daily work suffering? |  |  |
| 14 | Are you unable to play a useful part in life? |  |  |
| 15 | Have you lost interest in things? |  |  |
| 16 | Do you feel that you are a worthless person? |  |  |
| 17 | Has the thought of ending your life been on your mind? |  |  |
| 18 | Do you feel tired all the time? |  |  |
| 19 | Do you have uncomfortable feelings in your stomach? |  |  |
| 20 | Are you easily tired? |  |  |

**Part IV. World Health Organization-Disability Assessment Scale (WHODAS-12)**

This questionnaire asks about difficulties due to health conditions. Health conditions include diseases or illnesses, other health problems that may be short or long lasting, injuries, mental or emotional problems, and problems with alcohol or drugs.

Think back over the past 30 days and answer these questions, thinking about how much difficulty you had doing the following activities. For each question, please circle only one response. The information that you provide in this interview is confidential and will be used only for research.

Please Note: When scoring WHODAS, the following numbers are assigned to responses

1= No difficulty at all (None)

2 = Mild difficulty

3 = Moderate difficulty

4 = Severe difficulty

5 = Extreme difficulty or cannot do

|  | | In the past 30 days, how much difficulty did you have in: | None | Mild | Moderate | Severe | Extreme |
| --- | --- | --- | --- | --- | --- | --- | --- |
| 1 | | Standing for long periods such as 30 minute? | 1 | 2 | 3 | 4 | 5 |
| 2 | | Taking care of your household responsibilities? | 1 | 2 | 3 | 4 | 5 |
| 3 | | Learning a new task, for example, learning how to get to a new place? | 1 | 2 | 3 | 4 | 5 |
| 4 | | How much of a problem did you have joining in community activities (for example, festivities, religious or other activities) in the same way as anyone else can? | 1 | 2 | 3 | 4 | 5 |
| 5 | | How much have you been emotionally affected by your health problems? | 1 | 2 | 3 | 4 | 5 |
| 6 | | Concentrating on doing something for ten minutes? | 1 | 2 | 3 | 4 | 5 |
| 7 | | Walking a long distance such as a kilometer [or equivalent]? | 1 | 2 | 3 | 4 | 5 |
| 8 | | Washing your whole body? | 1 | 2 | 3 | 4 | 5 |
| 9 | | Getting dressed? | 1 | 2 | 3 | 4 | 5 |
| 10 | | Dealing with people you do not know? | 1 | 2 | 3 | 4 | 5 |
| 11 | | Maintaining a friendship? | 1 | 2 | 3 | 4 | 5 |
| 12 | | Your day-to-day work? | 1 | 2 | 3 | 4 | 5 |
|  | | | | | | | |
| H1 | Overall, in the past 30 days, how many days were these difficulties present? | | | | Record number of days ____ | | |
| H2 | In the past 30 days, for how many days were you totally unable to carry out your usual activities or work because of any health condition? | | | | Record number of days ____ | | |
| H3 | In the past 30 days, not counting the days that you were totally unable, for how many days did you cut back or reduce your usual activities or work because of any health condition? | | | | Record number of days ____ | | |

**Part V. Client Satisfaction Questionnaire**

Please help us improve our program by answering some questions about the services you have received. We are interested in your honest opinions, whether they are positive or negative.

Please answer all of the questions. We also welcome your comments and suggestions. Thank you very much; we really appreciate your help. Circle your answer:

| No | Question | Response | | | |
| --- | --- | --- | --- | --- | --- |
| 1 | How would you rate the quality of service you have received? | Excellent (4) | Good (3) | Fair (2) | Poor (1) |
| 2 | Did you get the kind of service you wanted? | No, definitely (1) | No, not really (2) | Yes, generally (3) | Yes, definitely (4) |
| 3 | To what extent has our program met your needs? | Almost all of my  needs have been met (4) | Most of my needs have been met (3) | Only a few of my needs have been met (2) | None of my needs have been met (1) |
| 4 | If a friend were in need of similar help, would you recommend our program to him or her? | No, definitely not (1) | No, I don’t think so (2) | Yes, I think so (3) | Yes, definitely (4) |
| 5 | How satisfied are you with the amount of help you have received? | Quite dissatisfied (1) | Indifferent or  Mildly dissatisfied (2) | Mostly satisfied (3) | Very satisfied (4) |
| 6 | Have the services you received helped you to deal more effectively with your problems? | Yes, they helped a  great deal (4) | Yes, they helped (3) | No, they really didn’t help (2) | No, they seemed  to make things  worse (1) |
| 7 | In an overall, general sense, how satisfied are you with the service you have received? | Very satisfied (4) | Mostly satisfied (3) | Indifferent or mildly  dissatisfied (2) | Quite dissatisfied (1) |
| 8 | If you were to seek help again, would you come back to our program? | No, definitely not (1) | No, I don’t think  So (2) | Yes, I think so (3) | Yes, definitely (4) |

**Topic guide for qualitatively measuring satisfaction of IPT-E**

1. How would you rate the quality of service you have received?
2. Did you get the kind of service you wanted?
3. To what extent has our program met your needs?
4. If a friend were in need of similar help, would you recommend our program to him or her?
5. How satisfied are you with the amount of help you have received?
6. Have the services you received helped you to deal more effectively with your problems?
7. In an overall, general sense, how satisfied are you with the service you have received?
8. If you were to seek help again, would you come back to our program?

# Part VI. Feasibility of IPT-E measuring questions

1. How many students consented to participate in the IPT-E intervention?
2. What is the number of students completed the intervention?
3. What is the attrition rate of the intervention?
4. What are the mean and modal numbers of session of the intervention?

**Part VII. Fidelity measuring instrument for counselors**

| IPT Treatment Checklist Prompt–  Patient chart number: ______________________________ Counselor________________________________  This Checklist can be completed by the counsellor for every session with each patient to help them use IPT-E. Please, tick your answer on the space provided. | | | | | | | | | | | | | | | | |
| --- | --- | --- | --- | --- | --- | --- | --- | --- | --- | --- | --- | --- | --- | --- | --- | --- |
| Items | Session 1 | | Session 2 | | Session 3 | | Session 4 | | Session 5 | | Session 6 | | Session 7 | | Session 8 | |
|  | Yes | No | Yes | No | Yes | No | Yes | No | Yes | No | Yes | No | Yes | No | Yes | No |
| Did you administer IPT-E screening tool? |  |  |  |  |  |  |  |  |  |  |  |  |  |  |  |  |
| Did you discuss psycho-social stressors which occurred around the same time as the symptoms started or worsened? |  |  |  |  |  |  |  |  |  |  |  |  |  |  |  |  |
| Did you ask about people in the patient’s life who may be helpful to them now? |  |  |  |  |  |  |  |  |  |  |  |  |  |  |  |  |
| Did you discuss the goal of treatment? |  |  |  |  |  |  |  |  |  |  |  |  |  |  |  |  |
| Did you discuss details of communication interactions or social role expectations in close relationships? |  |  |  |  |  |  |  |  |  |  |  |  |  |  |  |  |
| Did you use open questions and reflective, empathic statements to improve the patient’s experience of feeling understood by you? |  |  |  |  |  |  |  |  |  |  |  |  |  |  |  |  |
| Did you discuss ways to find or use people as social supports? |  |  |  |  |  |  |  |  |  |  |  |  |  |  |  |  |
| For grief, did you explore the events of the death, the relationship with the dead person, or ways to cope with the loss? |  |  |  |  |  |  |  |  |  |  |  |  |  |  |  |  |
| For role transitions did you explore the challenges of the patient’s new social role & what’s changed? |  |  |  |  |  |  |  |  |  |  |  |  |  |  |  |  |
| For disputes did you explore: the relationship with the disputed other; the issues in the disagreement; and identify problems and alternative ways to resolve misunderstandings? |  |  |  |  |  |  |  |  |  |  |  |  |  |  |  |  |
| Did you review the patient’s experience of treatment? |  |  |  |  |  |  |  |  |  |  |  |  |  |  |  |  |
| Did you discuss contingency plan in which the patient returns to the counseling office if they experience a relapse? |  |  |  |  |  |  |  |  |  |  |  |  |  |  |  |  |
| Did you ask if the patient have any worries or feelings about concluding treatment? |  |  |  |  |  |  |  |  |  |  |  |  |  |  |  |  |
