# Supplementary material for: Feasibility and acceptability of brief individual interpersonal psychotherapy among university students with mental distress in Ethiopia
Source: BMC Psychol. 2021 Apr 27;9:64. doi: 10.1186/s40359-021-00570-1 (PMC8077191; doi:10.1186/s40359-021-00570-1)
Supplement: Supplementary file 2 — Additional file 2. Treatment adherence and dose. [file 40359_2021_570_MOESM2_ESM.docx]

**Title of the manuscript:** Feasibility and Acceptability of Brief Individual Interpersonal Psychotherapy among University Students with Mental Distress in Ethiopia

**Author list:** Assegid Negash^12*^, Matloob Ahmed Khan^1^, Girmay Medhin^3^, Dawit Wondimagegn^1^, Clare Pain^4^ and Mesfin Araya^1^

^1^Department of Psychiatry, College of Health Sciences, School of Medicine, Addis Ababa University, Addis Ababa, Ethiopia

^*^ Correspondence: [assegidn@gmail.com](mailto:assegidn@gmail.com)

^2^Department of Psychology, College of Education and Behavioral Sciences, Wolaita Sodo University, Wolaita Sodo, Ethiopia

^3^Aklilu Lemma Institute of Pathobiology, Addis Ababa University, Addis Ababa, Ethiopia

^4^Department of Psychiatry, University of Toronto, Canada

Treatment adherence and dose

| Items | Session 1 | | Session 2 | | Session 3 | | Session 4 | | Session 5 | | Session 6 | | Session 7 | | Session 8 | |
| --- | --- | --- | --- | --- | --- | --- | --- | --- | --- | --- | --- | --- | --- | --- | --- | --- |
|  | Yes | No | Yes | No | Yes | No | Yes | No | Yes | No | Yes | No | Yes | No | Yes | No |
| Did you administer IPT-E screening tool and TTF? | 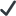 |  | 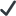 |  |  |  |  |  |  |  |  |  |  |  |  |  |
| Did you discuss psycho-social stressors which occurred around the same time as the symptoms started or worsened? |  |  | 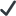 |  | 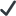 |  |  |  |  |  |  |  |  |  |  |  |
| Did you ask about people in the patient’s life who may be helpful to them now? |  |  | 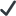 |  | 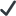 |  |  |  |  |  |  |  |  |  |  |  |
| Did you discuss the goal of treatment? |  |  | 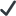 |  | 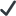 |  |  |  |  |  |  |  |  |  |  |  |
| Did you discuss details of communication interactions or social role expectations in close relationships? |  |  |  |  | 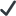 |  | 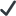 |  | 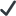 |  | 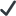 |  | 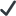 |  |  |  |
| Did you use open questions and reflective, empathic statements to improve the patient’s experience of feeling understood by you? |  |  | 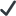 |  | 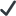 |  | 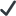 |  | 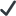 |  | 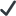 |  | 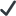 |  | 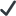 |  |
| Did you discuss ways to find or use people as social supports? |  |  |  |  | 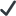 |  | 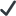 |  | 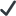 |  | 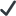 |  | 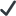 |  |  |  |
| For grief, did you explore the events of the death, the relationship with the dead person, or ways to cope with the loss? |  |  |  |  | 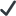 |  | 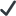 |  | 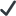 |  | 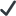 |  | 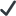 |  |  |  |
| For role transitions did you explore the challenges of the patient’s new social role & what’s changed? |  |  |  |  | 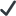 |  | 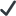 |  | 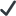 |  | 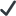 |  | 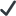 |  |  |  |
| For disputes, did you explore the relationship with the disputed other; the issues in the disagreement; and identify problems and alternative ways to resolve misunderstandings? |  |  |  |  | 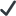 |  | 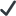 |  | 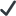 |  | 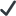 |  | 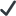 |  |  |  |
| Did you review the patient’s experience of treatment? |  |  |  |  |  |  |  |  |  |  |  |  |  |  |  | 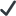 |
| Did you discuss contingency plan in which the patient returns to the counseling office if they experience a relapse? |  |  |  |  |  |  |  |  |  |  |  |  |  |  |  | 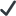 |
| Did you ask if the patient have any worries or feelings about concluding treatment? |  |  |  |  |  |  |  |  |  |  |  |  |  |  |  | 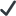 |
